# Treatment of Gonorrhœa with Tannin and Glycerine

**Published:** 1871-02

**Authors:** 


### TREATMENT OF GONORRHŒA WITH TANNIN AND GLYCERINE.

Dr. Schuster states that tannin with glycerine at first forms a soft waxy substance, which soon becomes hard and brown, and melts in a moist atmosphere at the temperature of the body. Dr. Schuster has formed small pencils of this compound, which he inserts into the urethra of patients suffering from gonorrhœa. He has found the treatment by means of caustic injections (the abortive method,) frequently to fail, and that it occasionally produces violent pain, inflammation and hemorrhage. On the other hand, the treatment with slighly astringent solutions cures the gonorrhœa within a period varying from four to seven weeks, but is often followed by a troublesome gleet. The tannin-glycerine rods employed by Dr. Schuster are from three to four inches in length, well rounded at the extremities, and consist of thirty grains of tannin, one grain of powdered opium, and a sufficient quantity of glycerine to form a pastille. These rods are hard in winter, and soft in summer. Before their introduction, they should be dipped in warm water. They are to be left for from five to ten minutes in the urethra, and then withdrawn. If, however, they be left in for an hour, or for a night. more or less pain is caused, and this appears to be due to a combination occurring between the tannin and the mucus or pus, which becomes hard and acts like a foreign body. Dr. Schuster has had no case of orchitis following the use of these pencils, though he has thought it advisable to recommend the employment of a suspensory bandage, nor has he noticed any irritation of the bladder or prostrate. In cases of gleet a rod may be left in for a few minutes; and speedy cure generally results.—*Lancet*, Oct. 8, 1870.

